# Supplementary figures and images for: Regulation of heme utilization and homeostasis in Candida albicans
Source: PLoS Genet. 2022 Sep 9;18(9):e1010390. doi: 10.1371/journal.pgen.1010390 (PMC9491583; doi:10.1371/journal.pgen.1010390)

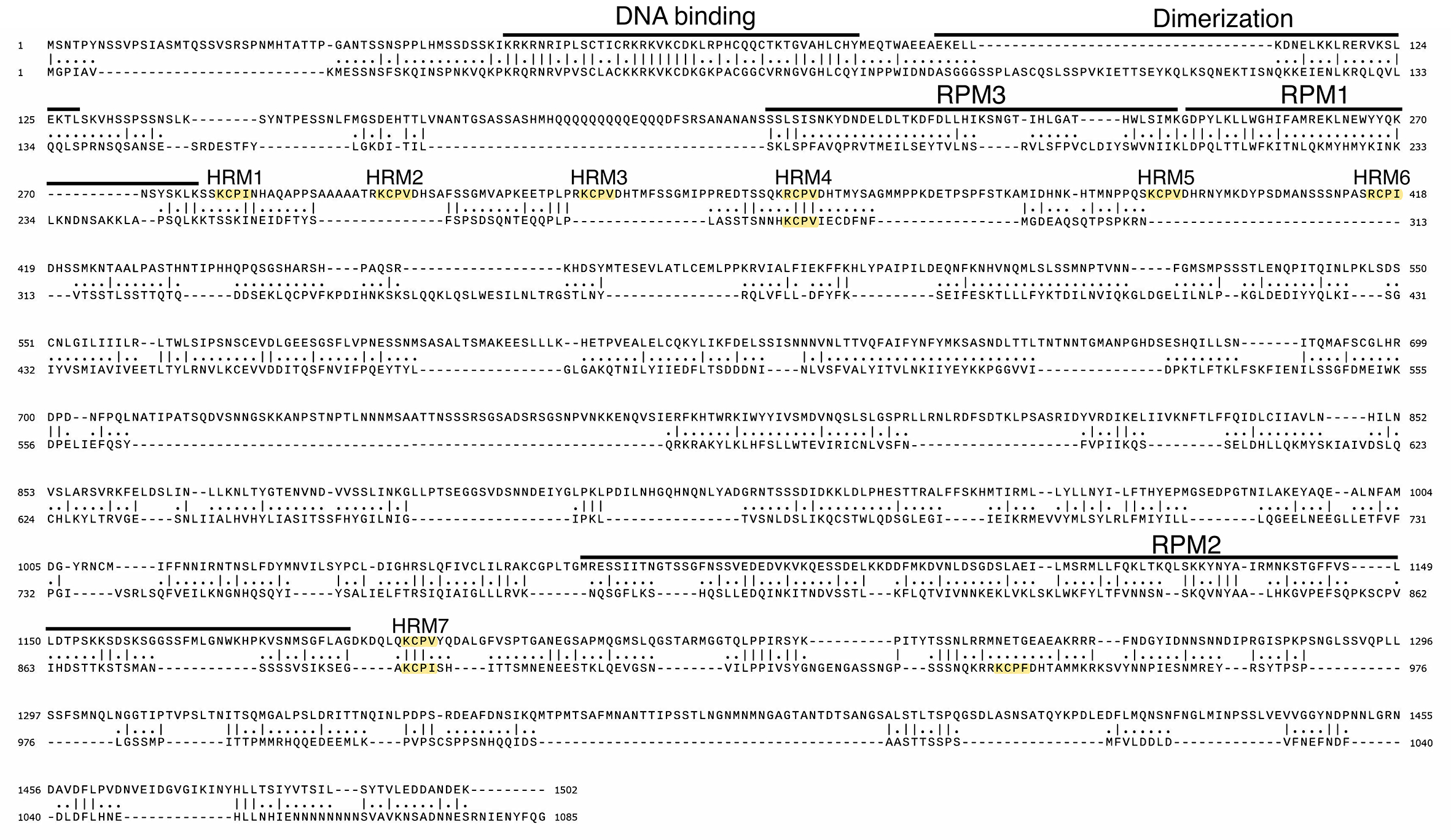

Supplement: S1 Fig — The S. cerevisiae Hap1 (top) was aligned with C. albicans Hap1/Zcf20 (bottom) using the MAFFT G-INS-i algorithm [63] with homologs. The DNA-binding and dimerization domains, the repressing modules (RPM1-3), and the heme-responsive motifs (HRM1-7) [33,35,64], of Hap1 are indicated above the sequence. The HRM consensus sequences R/KCPV/I in both proteins are highlighted. (JPG) [file pgen.1010390.s001.jpg]

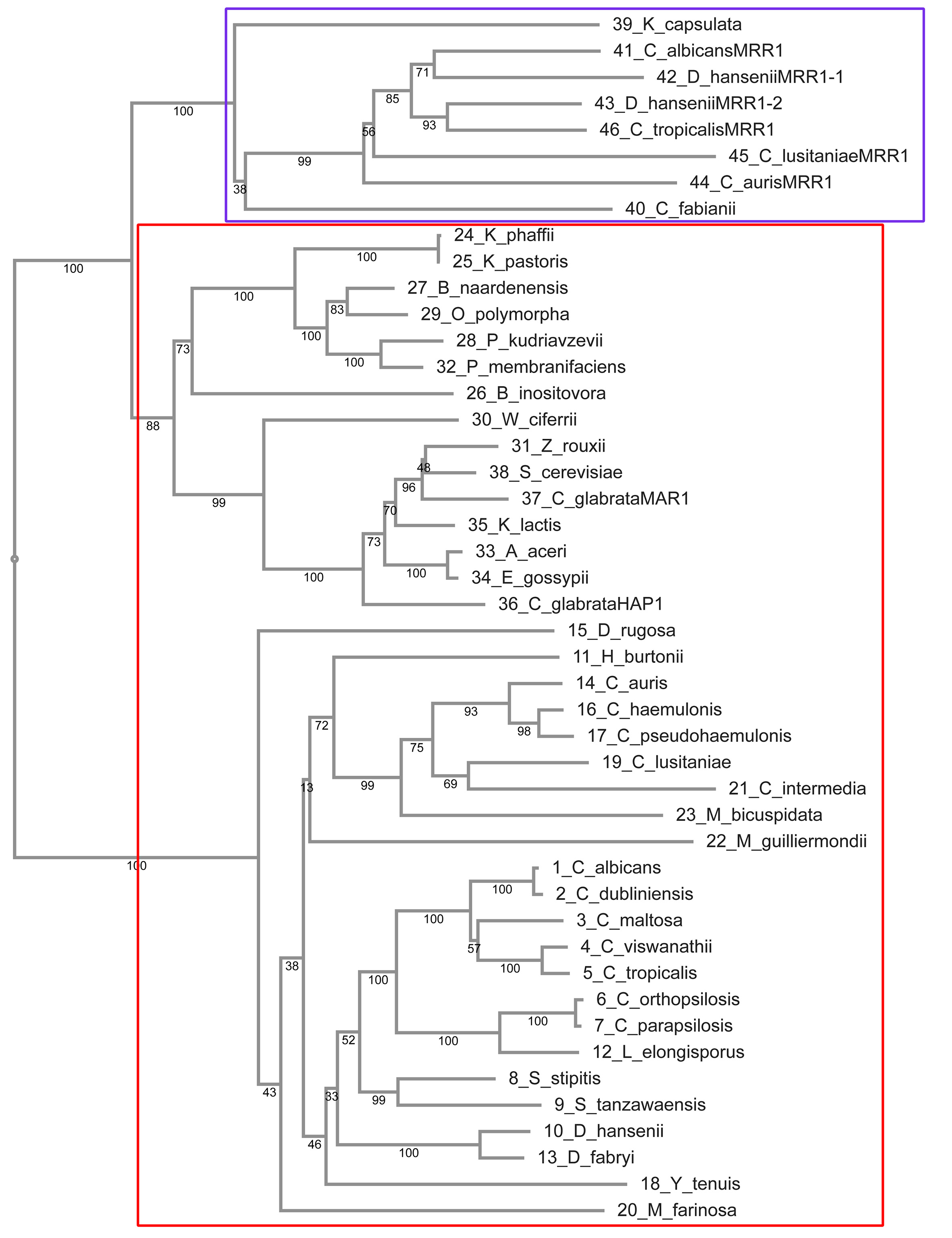

Supplement: S2 Fig — The Hap1 protein sequences of S. cerevisiae and C. albicans were used to identify the 44 most related sequences in 38 Saccharomycetales genomes and the sequences were aligned, followed by tree building. The red rectangle indicates sequences containing the HRM motif R/KCPV/I and the purple rectangle indicates sequences lacking it. (JPG) [file pgen.1010390.s002.jpg]

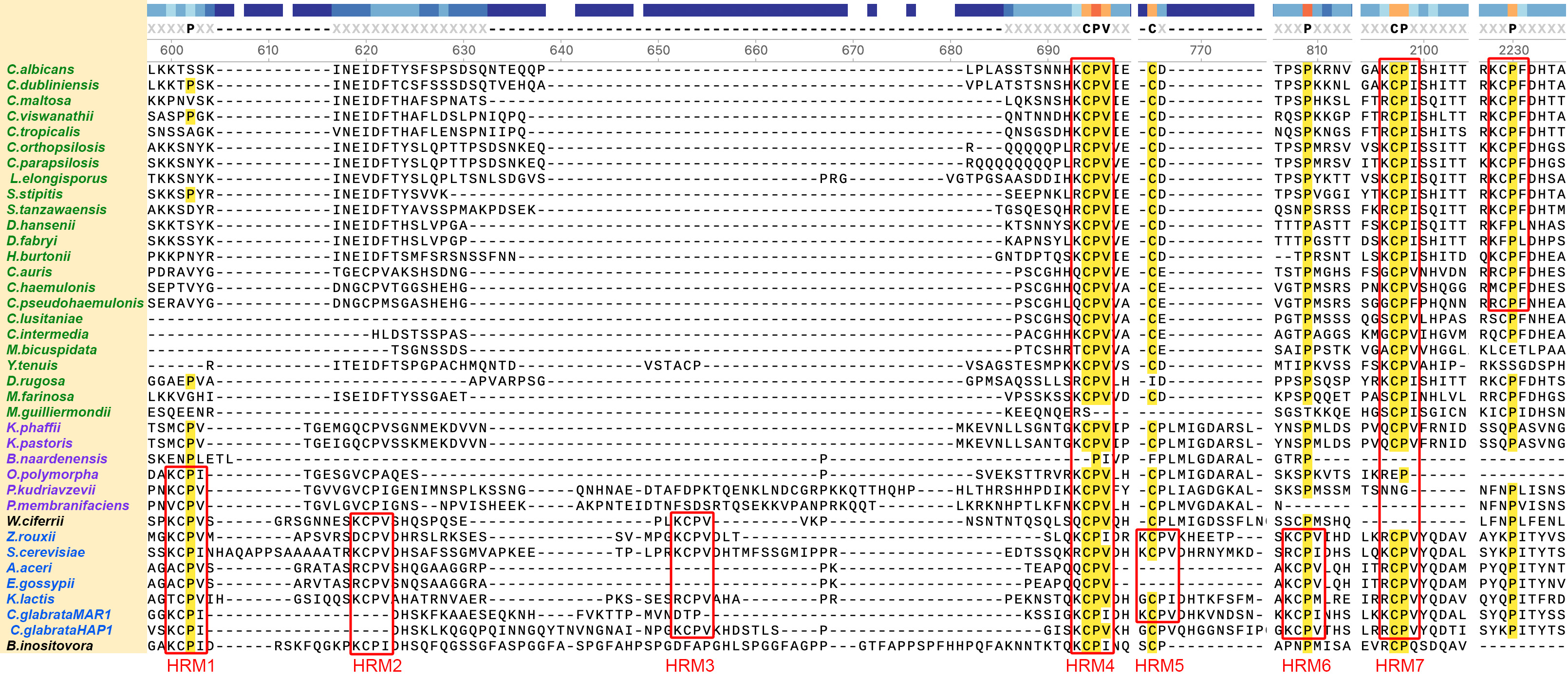

Supplement: S3 Fig — 38 Hap1 protein sequences identified by homology to the S. cerevisiae and C. albicans Hap1 sequences were aligned, and regions with conserved K/RCPV/I motifs were visualized (indicated with red rectangles). The S. cerevisiae Hap1 HRM1-7 motifs are indicated at the bottom. Residues conserved in more than half the sequences are highlighted. The species marked in green font belong to the CUG-Ser1 clade, the species marked in blue font belong to the Saccharomycetaceae and those in purple font, to the Pichiaceae. (JPG) [file pgen.1010390.s003.jpg]

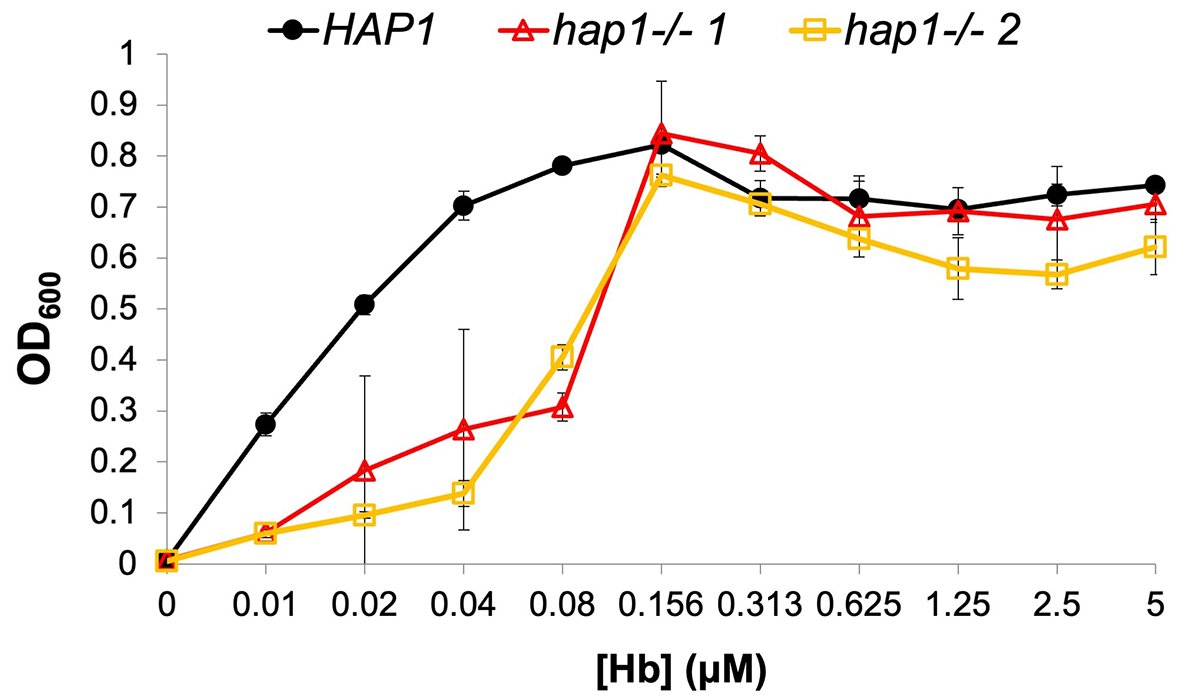

Supplement: S4 Fig — Two independent hap1-/- mutant clones from the Homann collection [36], together with wild-type strain SN148, were inoculated in desferrated RPMI medium supplemented with transferrin and with increasing amounts of hemoglobin, as indicated. The wild-type and mutant strains were inoculated in triplicate in 96 well plates, and incubated for 2 days at 30°C. The graph indicates the average density for each triplicate, and the error bars indicate the standard deviations. (JPG) [file pgen.1010390.s004.jpg]

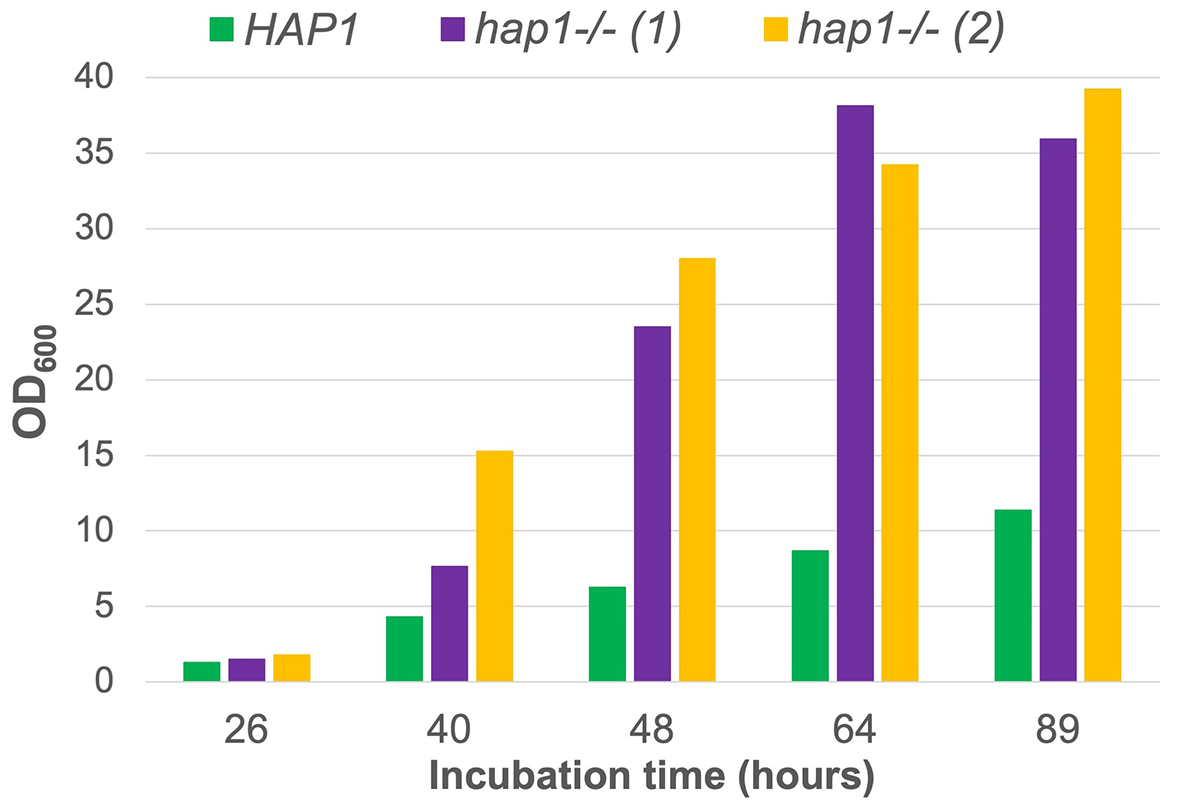

Supplement: S5 Fig — The wild-type strain SN148 and two independent hap1-/- mutant strains (KC1406) were inoculated at OD600 = 0.0005 in YPD supplemented with 1 mM ferrozine and 150 μM GaPPIX, and incubated at 30°C in 50 ml Falcon tubes with vigorous shaking. The densities were measured at the indicated times after inoculation. (JPG) [file pgen.1010390.s005.jpg]
