# Supplementary material for: Regulation of heme utilization and homeostasis in Candida albicans
Source: PLoS Genet. 2022 Sep 9;18(9):e1010390. doi: 10.1371/journal.pgen.1010390 (PMC9491583; doi:10.1371/journal.pgen.1010390)
Supplement: S7 Table — (DOCX) [file pgen.1010390.s012.docx]

**Table 3: List of primers**

| **#** | **Name** | **Sequence** |
| --- | --- | --- |
| **1** | F1-Transposo n-FPNI | CCGTTCGTT TTCGTTACCGGTATATC |
| **2** | F2-Transposo n-FPNI | CCGTCCCG CAAGTTAAATATG |
| **3** | F3-Transposon- FPNI | GTATTTTAC CGACCGTTACCGACC |
| **4** | R1-FPNI | CGCAGGTGACATAGAT GC |
| **5** | R2-FPNI | GACATAGA TGCTTAGCGCTGAGG |
| **6** | R3-FPNI | CGCAGGTG ACATAGATGCTTAGCG |
| **7** | PstI-HAP1 (+1) | GGCTGCAGATGGGTCCTATAGCTGTAA |
| **8** | HAP1-HindIII (+3255) | CCAAGCTTTCCTTGAAAGTAGTTTTCTATATT |
| **9** | SpeI- HAP1 (-1890) | GCGGCCGCTCTAGAACTAGTTCTAATAGCTTCCCACGGTATG |
| **10** | HAP1-HindIII (+3636) | TCGACGGTATCGATAAGCTTCTCAATATACTAGACCCTGG |
| **11** | HAP1-1 (-491 fwd) | GGATGTTATTTAGAATCAC |
| **12** | HAP1-3 (+24 rev) | cacggcgcgcctagcagcggGAAACTATTACTGCTTTCC |
| **13** | HAP1-4 (+3243 fwd) | gtcagcggccgcatccctgcCTACTTTCAAGGATAAATTTG |
| **14** | HAP1-6 (+3713 rev) | GTTCGATTTATAGGAAACAG |
| **15** | SpeI-HAP1 (+1258) | gcgactAGTTGAAAATCAGTGGGATTTA |
| **16** | HAP1-XhoI (+3255) | cgctcgagTCCTTGAAAGTAGTTTTCTATAT |
| **15** | ACT1f | TGAAGCCCAATCCAAAAGAGG |
| **16** | ACT1r | TTTCCATATCGTCCCAGTTGG |
| **17** | FRP2f | CGACAAGGTCAAAACAATGCG |
| **18** | FRP2r | GAGAAATGCCACTGAGTCAAATG |
| **19** | HAP1 60 fwd | ACCTAACAAAGTCCAGAAACCT |
| **20** | HAP1 145 rev | CGCATGCTGGTTTACCTTTATC |
| **21** | HMX1 fwd | GCACGATAGAGCAGACAAGACAG |
| **22** | HMX1 rev | TCCGGTTTCCAAAACTTGCTCC |
| **23** | PGA7 FWD | TTCCTCGATGCTTTCCACTGCC |
| **24** | PGA7 REV | ATGAGCCTGTAGATGACGAGCC |
| **25** | FRP1 FWD | GGAAACAAAGGAAGAATTGCCAC |
| **26** | FRP1 rev | CACCTTTCACCCTTCTAGCAC |
| **27** | RBT5f | TGCTCGCCTTATCCTTATTGTC |
| **28** | RBT5r | GTTGATGGAAGCGGTTTTAGC |
