# Supplementary material for: Regulation of heme utilization and homeostasis in Candida albicans
Source: PLoS Genet. 2022 Sep 9;18(9):e1010390. doi: 10.1371/journal.pgen.1010390 (PMC9491583; doi:10.1371/journal.pgen.1010390)
Supplement: Attachment — Submitted filename: HAP1 paper answer to reviewers.docx [file pgen.1010390.s015.docx]

*Dear Dr Kornitzer,

Thank you very much for submitting your Research Article entitled 'Regulation of heme utilization and homeostasis in Candida albicans' to PLOS Genetics.*

*The manuscript was fully evaluated at the editorial level and by independent peer reviewers. The reviewers appreciated the attention to an important topic but they identified some concerns that we ask you address in a revised manuscript. Most concerns van be addresses by clarifying the text.*

*Firstly, I would like you to reconsider naming the gene "Huf1". You have clearly shown that Huf1 is an ortholog of Hap1 in S. cereviaise, and the field can get very confused when different gene names are used in different species. Your manuscript is likely to appeal to a larger audience if it is clear that you are describing species-specific functions of Hap1.*

*Secondly, major new additional experiments suggested by the reviewers (such as confirming binding of Huf1, or overexpressing HUF1) are not necessary providing that the text is clear. You could consider assaying catalase as suggested by Reviewer 2. You should also clarify heme/hemin concentrations, confirm that the the number of replicates used is suitable, and clarify fold-changes discussed as suggested by the reviewers. Most other concerns can be addressed by changing some text, moving some material from supplementary to the main text, and for example referring to supplementary tables in the legend of Fig.6.*

We thank you and the reviewers for your comments on our manuscript. We have adopted your suggestion to rename Zcf20 Hap1 rather than Huf1. Your suggestion and Reviewer #2’s first comment prompted us to have a closer look at the homology between Zcf20 and Hap1. The sequences of Hap1 and Zcf20 are quite divergent, however comparison with related sequences from other yeasts, now shown in supplementary figures S1-S3, on the one hand, and the promoter analysis of Zcf20/hap1-dependent genes, and the heme sensitivity of the protein, on the other, convinced us that it would be more accurate to name the protein Hap1, as you suggest.

Our response to the reviewers’ comments appears below.

*Reviewer's Responses to Questions*

***Comments to the Authors:***

*Reviewer #1: (1) Line 160. Although the presence of the consensus ScHap1 in the promoter regions of some genes, there is no direct evidence that Huf1 directly binds to these promoters to promote transcription. This uncertainty should be mentioned in the text.*

Statistical evidence for the link between Hap1 and this consensus sequence is also presented in lines 294-303. To emphasize that no experimental evidence was obtained to show that Huf1 binds to this sequence, we added a sentence in the discussion: “The two proteins do nonetheless appear to have maintained a similar consensus binding site, based on its occurrence in the promoters of the most Hap1-dependent genes (Table 2), however direct binding of Hap1 to these sites remains to be shown experimentally..”

*(2) Something seems missing in the model shown in Figure 8. The coactivation of heme uptake and degradation is apparently not economical for the cell unless the main purpose of degradation is releasing iron for storage in addition to preventing heme toxicity. Figure 3 shows that the HMX1 expression level is at least 50 times higher than FRP2 in the absence of hemin. How will these data fit into the model?*

Regarding HMX1 vs. FRP2 transcript levels, the NGS-derived data may be more directly comparable, and they indicate a smaller difference, of 10-fold. But we are not sure we understand the concern regarding this difference. We don’t have a way to predict what the relative baseline levels of these transcripts should be, since they will depend on the relative activity of Hap1 on each promoter, the activity of other transcription factors, post-transcriptional effects that affect e.g. transcript stability, etc.

Regarding the first comment, we agree that the feedback loops that promote heme uptake and heme degradation might, once the cellular heme and iron requirements are met, cause iron overload if not kept in check. We have added mention of this possibility when describing the model in the Discussion.

*Reviewer #2: This manuscript describes a genetic dissection of the Huf1 transcription factor of Candida albicans. At the beginning, the authors used a stable haploid C. albicans strain that was mutagenized with the Ac/Ds transposon to isolate GaPPIX-resistant mutants. Based on their reduced ability to grow on hemoglobin as the sole iron source, several mutants were characterized for their transposon insertion site. Among them, the authors identified that inactivation of the HUF1 gene in a standard diploid C. albicans strain recapitulates the earlier phenotype. Using fluorescent heme sensors, the absence of Huf1 results in higher cytoplasmic heme concentrations. The authors establish that HUF1 is transcriptionally induced in the presence of hemin. They show that Huf1 is a nuclear protein that is stabilized in the presence of hemin. The authors used RNA-Seq to investigate the transcriptional response under hemin-replete conditions and identified several genes that exhibit Huf1-dependent changes on a genome-wide scale. Overall, the findings will certainly be of interest to the field of microbial heme homeostasis and fungal pathogens.

Comments.

1. A schematic illustration of C. albicans Huf1 with Saccharomyces cerevisiae Hap1 would be important to add as a figure in the manuscript. In this way, location of conserved domains such as DNA-binding and heme-binding domains of these transcription factors could be shown. Percentages of identity and similarity between these two proteins would also be very informative.*

We have added an extensive analysis of the similarities between *C. albicans* Hap1 and *S. cerevisiae* Hap1, as well as related sequences from other yeasts. This is now shown in supplementary figs. S1-S3. *2. Is there any experimental evidence that Huf1 or a portion of Huf1 directly binds hemin? Is there absorbance spectroscopy that has been performed to detect changes at the Soret peak?*

No. Unfortunately, our attempts to express *C. albicans* Hap1 in either *E. coli* or *Pichia* were not successful.
 *3. According to the proposed model (Fig. 8), Huf1 activates genes encoding proteins involved in heme acquisition and degradation. Do the authors have performed ChIP analysis to test whether Huf1 exhibits differential binding as a function of the presence or absence of hemin and/or iron availability?*

We did not directly test binding of C. albicans Hap1 to the promoter sequences.
 *4. The authors conclude that the DNA binding motif recognized by Huf1 is to some extent similar to that of Hap1, which binds to a DNA sequence CGGNNNTANCGG. Do the authors have experimental data to support this conclusion? Do the authors have used promoter-lacZ fusions to map cis-acting elements necessary for hemin- and Huf1-dependent activation of gene expression?*

There is no direct experimental evidence that *C. albicans* Hap1 binds to the Hap1 consensus sequence. The argument is statistical, based on the occurrence of this motif in the promoter of genes that are regulated by *C. albicans* Hap1. This fact is emphasized in a sentence we added to the discussion: “The two proteins do nonetheless appear to have maintained a similar consensus binding site, based on its occurrence in the promoters of the most Hap1-dependent genes (Table 2), however direct binding of Hap1 to these sites remains to be shown experimentally.”

*5. Does overexpression of Huf1 enhance hemin or heme analog acquisition? Does overexpression of Huf1 lower cytoplasmic heme levels through measurement using cytoplasmic fluorescent heme sensors?*

We did not test the effect of *C. albicans* Hap1 overexpression on hemin acquisition or cytoplasmic heme levels.
 *6. In Fig. 4, what would happen if wild-type HUF1 is returned in huf1-/- cells? Does it restore cytoplasmic heme to wild-type levels? In huf1-/- cells, cytoplasmic heme levels are higher, do huf1-/- cells exhibit normal catalase activity? This would be very interesting to assess since in Fig. 6B, the CAT1 gene (encoding catalase) appears to be a Huf1 target gene.*

Regarding the effect of reintegrating the *C. albicans* *HAP1* gene into the *hap1* mutant, this was tested in some of the assays, where the reintegrant was indeed found to exhibit the same phenotype as the *HAP1* wild-type strain – Figs 1A-C, 2A – indicating that the heme-related phenotypes of the *hap1* mutant are due to deletion of *HAP1*. The other way to show this was to test two independently constructed hap1 deletion strains and satisfy ourselves that they exhibit the same phenotype, distinct from the wild-type. This was done in Figs. 1A, B, 2B, and Fig. 3, in addition to Fig. 4.

Regarding the catalase activity: although heme levels are higher in the *hap1*-/- strain, and catalase is a heme enzyme, there is no reason to think that heme is limiting for catalase biogenesis in the wild-type. As the reviewer pointed out, *CAT1* transcript levels were indeed lower in the *hap1* mutant – specifically, 3x lower in the presence of hemin (Table 2) and 1.9x lower in regular YPD medium (Table S1). When we tested catalase activity (ΔOD280/mg/min with 10 mM H_2_O_2_ as substrate), we found the following results with the hap1 mutant (KC1408) vs. the HAP1 reintegrant (KC1407) grown 4 h in YPD or in YPD + 50 μM hemin:

*HAP1*: 0.030 +/- 0.007 ; *hap1*-/-: 0.020 +/- 0.002;

*HAP1* + hemin: 0.029 +/- 0.003 ; *hap1*-/- + hemin: 0.015 +/- 0.000

Thus the lower expression of *CAT1* in the *hap1*-/- mutant is mirrored in the lower catalase activity of these cells, both without and with added hemin. Although these results provide support for the *CAT1* expression data, we do not feel that they add enough information to justify adding them to the manuscript.

*7. In the text that describes results in Fig. 4, especially in lines 187 to 212, fold values are not indicated and are not described with enough detail. These data should be commented with more detail since without a clear description, it is very difficult to understand and therefore appreciate the results.*

Thanks for pointing this out. We have expanded the description of these results in the text, including mention of the relative decrease in fluorescence ratios where relevant. *8. In Fig S3 and Fig 5, why are three different concentrations of hemin that were used? Experiments in Fig S3 were performed using 10 uM hemin, whereas experiments in Fig 5 A and C were performed using 50 uM and 20 uM hemin, respectively. How could you compare the data if different hemin concentrations have been used?*

With regard to 50 μM hemin, lower concentrations are sufficient to induce nuclear localization (Fig. S3, now 5B) however for the metal-substituted heme analogs (Fig. 5B), higher concentrations were required. We tested different concentrations, 50 μM was the concentration best suited to show the relative effect of the different metal-substituted heme analogs. With regard to the stabilization (Fig. 5C), we started by testing 20 μM and since it showed a clear stabilization in two experiments, we did not test other concentrations. However please note that we do not directly compare the localization and decay experiments since they involve different tags, different promoters and different media. We have moved mention of compound concentrations from the figure itself to the legend to reduce the impression that these experiments are directly comparable. *9. In Fig 5B, it is unclear whether there is significant increase of the nuclear Huf1-GFP signal in the presence of GaPPIX and MnPPIX as compared with untreated cells? In the case of GaPPIX, it is intriguing that the signal is so low since the original screen was performed to identify GaPPIX-resistant clones. I would recommend to specify that no signal is seen in the presence of MnPPIX.*

Thank you for pointing this out. We agree that the signal in this concentration of MnPPIX is so low as to qualify as “no signal”. We do mention that now in the text. With regard to the relatively low Hap1-GFP level in the presence of 50 μM GaPPIX, vs. the fact that *HAP1* was identified in a GaPPIX-based screen, please note that for the screen, 150 μM GaPPIX was used.
 *10. In Fig. 5C, only three time points have been taken after glucose addition, with only a single time point (90 min) that indicates a significant difference. It would be very informative to take additional measurements as a function of time (longer time points like Fig 7B in which 2, 4, and 6 h time points have been taken), especially for assessment of the protein levels of Huf1 in the absence or presence of hemin.*

Where protein degradation is concerned, we believe that 90 min is quite long already – note that we are following the decay of a bolus of proteins made before the shutoff of the MAL2 promoter, rather than monitoring a cellular state at different times after induction as in other assays. However, the point is well taken that a single datapoint is not optimal to support our conclusion of Hap1 stabilization by hemin. Since the experiment was performed twice, we are now showing the average of the two experiments in the graph, with SD. We believe this provides much better support for our conclusion.
 *11. Fig. 6B would beneficiate to be shown as regular Table(s) in the manuscript to list and describe all target genes. The current Venn diagram with color codes is very difficult and confusing to understand for the reader.*

We would prefer to leave this figure, as other readers have found it helpful. But the point is well taken. The different intersects of this figure are now presented as lists in a new sheet of Supplementary Table S2 and we refer to it in the legend to Fig. 6.

*Reviewer #3: This is an excellent manuscript demonstrating for the first time, a heme regulated transcription factor in the opportunistic fungal pathogen Candida albicans. While a great deal is understood regarding the complex control of C. albicans Fe homeostasis through the SFU1-SEF1-HAP43 regulatory network, this is the first demonstration of a separate transcription factor dedicated to heme uptake. Most interesting, the ortholog of this factor in the non-pathogenic S. cerevisiae does not participate in heme uptake as heme is not an abundant source of Fe for this organism. The repurposing of this transcription (now called HUF1 in C. albicans) is a clever adaptation of the pathogenic yeast to existence in an animal host. The paper is rigorous, comprehensive and written nicely for a diverse audience. I only have a few relatively minor suggestions for improvement.

1. Statistics are needed for Fig. 4A and Fig. 4B. The authors discuss differences in the huf1 mutant versus WT strain that are not obvious in the graphs provided without the appropriate analysis to show these changes are statistically significant. In the same figure, the authors say the results are the averages of three cultures. Does this mean the experiment was done only once with three cultures?*

The experiments were done with three (Fig. 4A) or four (Fig. 4B) cultures for each strain. Statistical analysis was added to the figure. To simplify the figure, the non-heme binding control, DM, which is not very informative, was removed.  *2. In the legend to Fig. 4, it would be helpful to define HS1 (heme sensor), M7A (lower heme affinity probe) and DM (non heme binding control).*

Done. *3. Lines 221 – 224 and line 369 mention “regular media”. What does "regular" mean, specifically in terms of available iron and heme sources?*

We have now specified the media conditions in these sentences. *4. Is it possible to insert the data of Fig. S3 into main Fig. 5. The title of the section is “Huf1 is nuclear localized…” but one has to download supplemental material to see the data that support this important claim.*

We have moved this panel to Fig. 5 as suggested.
